# Reduction of orexin-expressing neurons and a unique sleep phenotype in the Tg-SwDI mouse model of Alzheimer’s disease

**DOI:** 10.3389/fnagi.2025.1529769

**Published:** 2025-02-04

**Authors:** Yan Wu, Narayan R. Bhat, Meng Liu

**Affiliations:** ^1^Department of Psychiatry and Behavioral Sciences, Medical University of South Carolina, Charleston, SC, United States; ^2^Department of Neuroscience, Medical University of South Carolina, Charleston, SC, United States

**Keywords:** sleep, Alzheimer’s disease, orexin, cerebral amyloid angiopathy, Tg-SwDI, apoptosis

## Abstract

Sleep disturbances are common in Alzheimer’s disease (AD) and AD-related dementia (ADRD). We performed a sleep study on Tg-SwDI mice, a cerebral amyloid angiopathy (CAA) model, and age-matched wild-type (WT) control mice. The results showed that at 12 months of age, the hemizygous Tg-SwDI mice spent significantly more time in non-rapid eye movement (NREM) sleep (44.6 ± 2.4% in Tg-SwDI versus 35.9 ± 2.5% in WT) and had a much shorter average length of wake bout during the dark (active) phase (148.5 ± 8.7 s in the Tg-SwDI versus 203.6 ± 13.0 s in WT). Histological analysis revealed stark decreases of orexin immunoreactive (orexin-IR) neuron number and soma size in these Tg-SwDI mice (cell number: 2187 ± 97.1 in Tg-SwDI versus 3318 ± 137.9 in WT. soma size: 109.1 ± 8.1 μm^2^ in Tg-SwDI versus 160.4 ± 6.6 μm^2^ in WT), while the number and size of melanin-concentrating hormone (MCH) immunoreactive (MCH-IR) neurons remained unchanged (cell number: 4256 ± 273.3 in Tg-SwDI versus 4494 ± 326.8 in WT. soma size: 220.1 ± 13.6 μm^2^ in Tg-SwDI versus 202.0 ± 7.8 μm^2^ in WT). The apoptotic cell death marker cleaved caspase-3 immunoreactive (Caspase-3-IR) percentage in orexin-IR neurons was significantly higher in Tg-SwDI mice than in WT controls. This selective loss of orexin-IR neurons could be associated with the abnormal sleep phenotype in these Tg-SwDI mice. Further studies are needed to determine the cause of the selective death of orexin-IR cells and relevant effects on cognition impairments in this mouse model of microvascular amyloidosis.

## Introduction

The bi-directional relationship between sleep and Alzheimer’s disease (AD) and AD-related dementia (ADRD) has become a focus of recent AD research (Peter-Derex et al., 2015, Reddy and van der Werf, 2020). Aβ clearance from the brain’s ISF (interstitial fluid) predominately occurs during sleep via glymphatic efflux (Iliff et al., 2012). The concentration of soluble amyloid decreases during sleep while it increases during waking. One night of sleep deprivation can induce significant Aβ accumulation in the human brain (Kang et al., 2009, Shokri-Kojori et al., 2018). The sleep/wake cycle and orexin (hypocretin), a wake-promoting neuropeptide, regulate Aβ dynamics (Fronczek et al., 2012). Elderly patients with narcolepsy, a sleep disorder due to a deficiency of wake-promoting neuropeptide orexin (hypocretin, HCRT), have reduced Aβ burden (Gabelle et al., 2019). Orexin gene knockout mice show decreased Aβ deposition (Roh et al., 2014). These lines of evidence suggest that sleep might be critical in Aβ accumulation and AD pathogenesis. Increasing or consolidating sleep showed beneficial effects on AD pathology, while disrupted sleep or prolonged waking could exacerbate AD pathology (Ju et al., 2017, Wu et al., 2019, Tekieh et al., 2022).

Conversely, sleep disruption is also the primary non-cognitive symptom in human AD and ADRD. The typical sleep disturbances in AD and ADRD patients include difficulty sleeping, sleep fragmentation, reduced amount of non-rapid eye movement (NREM) sleep (NREMS) or slow-wave sleep and reduced EEG delta (δ) power, though significant sleep loss is absent (Peter-Derex et al., 2015, Borges et al., 2019, Lucey et al., 2021, Yuan et al., 2022, Falgas et al., 2023, Koemans et al., 2023). Sleep disturbances usually get worse as dementia progresses in severity, and excessive sleepiness becomes common in later-stage dementia (Elwood et al., 2011, Cavailles et al., 2022). Many of these abnormal sleep phenotypes, such as sleep fragmentation and reduced NREM sleep amount and intensity, have been replicated in mouse models of AD that include APP/PS1, 3xTg-AD, Tg2576, J20, APP23, and 5xFAD mice (Sethi et al., 2015, Kent et al., 2018, Van Erum et al., 2019, Filon et al., 2020, Drew et al., 2023). These models exhibit exclusive or predominant parenchymal Aβ deposition or tauopathy. Tg-SwDI (APP-Swedish-Dutch-Iowa) mice is a capillary cerebral amyloid angiopathy (capCAA) model that expresses Aβ deposits primarily in the cerebral microvasculature and relatively mild in brain parenchyma at specific age ranges (Davis et al., 2004, Park et al., 2014, Xu et al., 2014). To our knowledge, the sleep phenotypes of Tg-SwDI mice have not been reported. We performed a sleep study in 11∼12-month-old Tg-SwDI mice and identified a unique sleep pattern and potential neural substrate mediating these sleep changes in these mice.

## Materials and methods

### Animals and surgery

Animal breeding and manipulations followed the policies established in the National Institutes of Health Guide for the Care and Use of Laboratory Animals and the Institutional Animal Care and Use Committee (protocol # IACUC-2021-1399).

Six (both sexes) 11∼12-month-old Tg-SwDI mice (Jax # 034843, hemizygotes) and five (both sexes) age-matched C57BL/6 WT mice were used in this sleep study. Additional 12-month-old homozygous Tg-SwDI mice and 5xFAD mice (Jax # 034848) were added to compare the types and distribution patterns of Aβ deposits and the numbers of orexin and MCH neurons in Supplementary material.

Genotype validation on mice tail snips was done off-site by Transnetyx (Cordova, TN). All mice were housed in a 12 h/12 h light/dark environment with lights on at zeitgeber time 0 (ZT00) and fed ad libitum standard rodent chow and water. Ambient temperature and humidity were maintained between 22 and 24°C and 40–60%, respectively.

Under deep anesthesia (isoflurane 1.0–2.0%) and using a stereotaxic frame (Kopf, Tujunga, CA), and as described elsewhere (Liu et al., 2011), two small screw-type electrodes (Protech, CA) were implanted onto the frontal and parietal mouse skull for recording the electroencephalogram (EEG). A pair of plate-type electrodes (Protech, CA) were inserted into nuchal muscles for electromyogram (EMG) activity recording. Three weeks after surgery, mice were individually housed in the recording cages and connected to the lightweight EEG/EMG cables for 3-day acclimation. Then, 24-h EEG/EMG signals were acquired after being amplified and filtered (0.3–100 Hz for EEG; 100-1K Hz for EMG, MP150 system; Biopac Systems Inc., CA).

### Sleep data scoring and EEG power analysis

EEG/EMG data were scored in 10-second epochs as wakefulness, NREM sleep, and rapid eye movement (REM) sleep (REMS) with SleepSign software (KISSEI Comtec Ltd., Nagano, Japan). Wakefulness is identified by the presence of desynchronized EEG coupled with high-amplitude EMG activity. NREM sleep is identified when the EEG shows high-amplitude/low-frequency waves ( < 4 Hz, delta waves) together with a lower EMG activity relative to waking. REM sleep is identified by the presence of regular EEG theta activity (4–8 Hz) coupled with very low EMG activity. The percentage of time spent in each sleep/vigilance stage and the average length of wake bouts were calculated, and EEG power spectral analysis was performed using the Fast Fourier Transform (FFT) built into the SleepSign software after all artifacts were removed. All power data was normalized as the percentage of the total power from 0 to 40 Hz (Kent et al., 2018, Sun et al., 2019, Van Erum et al., 2019).

### Histology

The day after the sleep recording, all mice were anesthetized with isoflurane (overdose) and perfused transcardially with 0.9% PBS (5–10 mL), followed by 10% buffered formalin in 0.1M PBS (50 mL) between Zeitgeber time (ZT) 14:00–15:00. Mice brains were harvested and cross-sectioned at 40 μm thickness on a compresstome (Precisionary Instruments, Greenville, NC). Sections were divided evenly into four sets, with one set stained with mouse anti-orexin-A monoclonal antibody (1:1,000 dilution, SC-80263, Santa Cruz Biotech, CA) and goat anti-pro-MCH polyclonal antibody (1:1,000 dilution, SC-14509, Santa Cruz Biotech, CA) for counting orexin and MCH immunoreactive cells. The other set of sections was selected for Thioflavin-S (ThioS, 0.5%) staining to show fibrillar Aβ deposits/plaques (Schmidt et al., 1995, Bussiere et al., 2004), followed by double-labeled immunofluorescent stainings using the following antibodies: goat-anti-CD31 polyclonal antibody (1:200 dilution, AF3628, Novus Biologicals, Centennial, CO) to show vasculature (blood vessels) (Wang et al., 2022) and mouse anti-human Aβ monoclonal antibody (1:1,000 dilution, 82E1-Biotin, IBL-America, Minneapolis, MN) to show both nonfibrillar and fibrilar Aβ deposits. The third set of sections was co-stained for either orexin and cleaved caspase-3 (CCasp3) or pro-MCH and CCasp3 (1:500 dilution, 7961, Cell Signaling Tech, Danvers, MA) to detect cell death. Alexa Fluor streptavidin-568 was used to detect Biotin in 82E1-Biotin immunostaining; Alexa Fluor-488, -568, and -647 donkey IgGs were used as the secondary antibodies for other immunostainings (1:500 dilution, Thermofisher, Waltham, MA). High-definition images from immunostaining and ThioS staining results were taken using a Leica TCS SP8 confocal microscope for cell counting and image analysis.

### Orexin immunoreactive (orexin-IR) and MCH-immunoreactive (MCH-IR) cell quantification

Confocal images of immunostaining were analyzed using NIH ImageJ software (Version 1.53). Images from 8 to 10 brain sections containing orexin-IR and MCH-IR cells were converted to binary images with black or white pixels and calibrated. Then, the numbers and average soma areas of orexin-IR/MCH-IR cells were measured using the “particle analyze” function with a predetermined threshold value. The experimenters performing the morphometry were blind to the group identification of each section and used the same threshold and brightness for all images analyzed. Only immunoreactive cells containing a contour of the nucleus were counted and measured to avoid overestimation. The total numbers of orexin/MCH cells in each mouse were reported as the sum of bilateral counts of immunoreactive cells multiplied by four.

### Statistical analysis

Data were analyzed using GraphPad Prism 9.2 (GraphPad Software, Boston, MA). Time spent in each stage, average wake bout length, and EEG power during the active (dark) and inactive (light) phases were compared between Tg-SwDI mice and the WT mice with two-way ANOVA combined with Bonferroni post-hoc test. Unpaired t-tests were used to compare orexin-IR/MCH-IR cell numbers and average soma size between WT and Tg-SwDI mice. All values were expressed as Mean ± SEM, and the statistical significance was evaluated at the *p* < 0.05 level.

## Results

### Hemizygous Tg-SwDI mice at 12 months old are a primary CAA model with predominant microvascular Aβ deposits in the brain

We used the Thioflavin-S (ThioS) to stain fibrillar Aβ deposits/plaques and a biotinylated mouse monoclonal antibody against human Aβ (82E1-Biotin) to stain both nonfibrillar and fibrillar Aβ deposits. The 82E1-Biotin antibody was chosen to prevent non-specific staining, commonly called “mouse on mouse” (MOM), which can occur when using a mouse primary antibody on mouse tissue. Additionally, a polyclonal antibody against CD31 was used to stain blood vessels.

**FIGURE 1:**
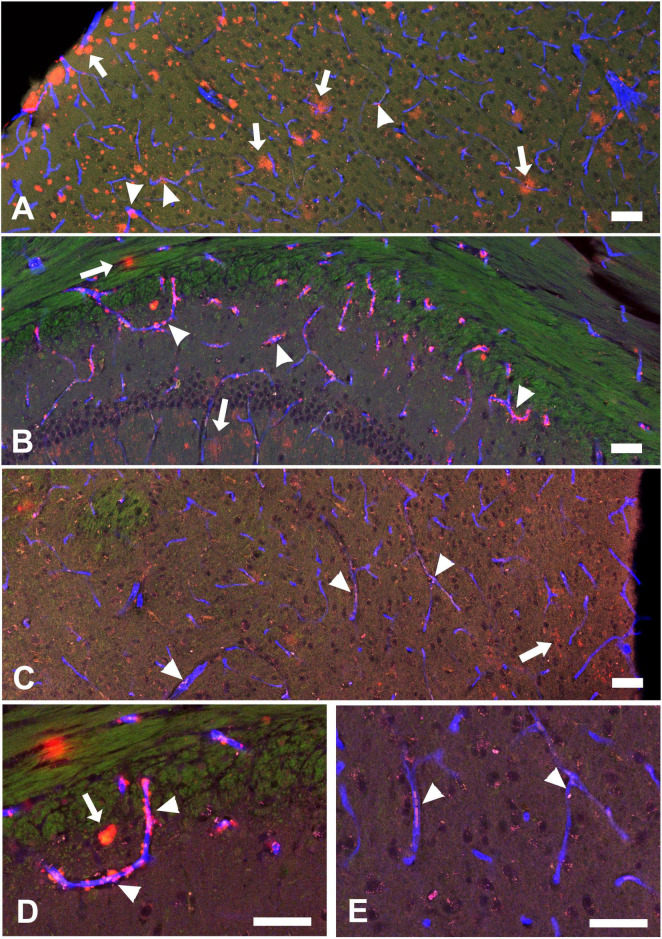
Aβ deposit distributions in the cortex **(A)**, hippocampal CA1 area **(B)**, and hypothalamus **(C)** in a 12-month-old hemizygous Tg-SwDI mouse. Green: Thioflavin-S (ThioS) staining for Aβ plaques, Red: Aβ (82E1) staining for Aβ deposits, Blue: CD31 staining to show blood vessels. These brain regions exhibit weak or negative ThioS^+^ staining but strong 82E1^+^ Aβ deposits. **(D,E)** Higher magnification images detailing Aβ distribution in hippocampal CA1 and hypothalamus. Arrows indicate parenchymal Aβ (82E1^+^/CD31^–^), while arrowheads highlight vascular Aβ (82E1^+^/CD31^+^). Vascular Aβ deposits could be observed inside the blood vessels or surrounding blood vessel walls (perivascular Aβ). Scale bar = 50 μM.

In our Tg-SwDI mice, ThioS positive (ThioS^+^) signals were primarily observed in the septum nucleus and dentate gyrus, with minimal or no presence in other brain regions. Diffuse Aβ deposits, revealed by 82E1 staining, were present in many brain regions and were mostly nonfibrillar (82E1^+^/ThioS^–^). Some Aβ deposits were located within or around blood vessels outlined by CD31 staining, indicating these were vascular or perivascular deposits. In the lateral hypothalamus, where orexin and MCH neurons reside, ThioS staining was negative, and the overall intensity of 82E1^+^ signals was less intense, but there were clear vascular Aβ deposits and weakly stained parenchymal Aβ deposits (Figure 1). ThioS and 82E1 staining results were negative in wild-type (WT) mice.

In summary, at 12 months of age, hemizygous Tg-SwDI mice displayed a characteristic cerebral amyloid angiopathy (CAA) pattern, marked by robust microvascular Aβ deposits and mild to moderate parenchymal Aβ deposits. Supplementary Figure S1 illustrates the distinct Aβ distribution in 12-month-old homozygous Tg-SwDI mice. Quantitative comparisons of vascular and parenchymal Aβ deposits between hemizygous and homozygous Tg-SwDI mice are presented in Supplementary Figure S2. At 12 months of age, the homozygotes exhibit more extensive parenchymal Aβ deposits and fewer vascular Aβ deposits than hemizygotes. Furthermore, Aβ deposits in homozygotes are predominantly fibrillar (ThioS^+^ plaques), whereas those in hemizygotes are primarily nonfibrillar (ThioS^–^).

### Increased NREM sleep, shortened wake bouts, and shifted theta power spectra in Tg-SwDI mice

**FIGURE 2:**
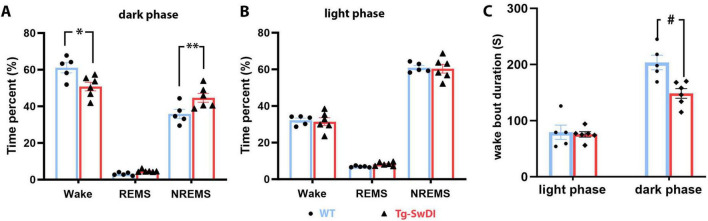
Results of the sleep study. **(A)** Tg-SwDI mice spent significantly more time in NREM sleep than the WT mice by sacrificing time spent in waking during the active (dark) phase (**A**, *p = 0.017. **p = 0.0045). **(B)** Time spent in each vigilance state during the inactive (light) phase was indifferent between the two groups. **(C)** The average length of wake bouts during the active (dark) phase was significantly shortened in Tg-SwDI mice (#*p* = 0.0019).

Sleep data analysis showed that 12-month-old hemizygous Tg-SwDI mice exhibited significant sleep/wake alterations during the dark (active) phase compared to age-matched WT mice. Specifically, Tg-SwDI mice spent significantly more time in NREM sleep during the dark phase (44.64 ± 2.41% in Tg-SwDI versus 35.86 ± 2.50% in WT, *p* = 0.017, *t* = 3.02, *df* = 27), which came at the expense of time spent in waking (50.78 ± 2.37% in Tg-SwDI versus 61.07 ± 2.75% in WT, *p* = 0.0045, *t* = 3.54, *df* = 27) (Figures 2A,B). Additionally, the average length of wake bouts during the dark phase was significantly shorter in Tg-SwDI mice (148.50 ± 8.70 s versus 203.60 ± 13.03 s in WT, *p* = 0.0019, *t* = 3.94, *df* = 18), suggesting a sleep/wake fragmentation (Figure 2C).

**FIGURE 3:**
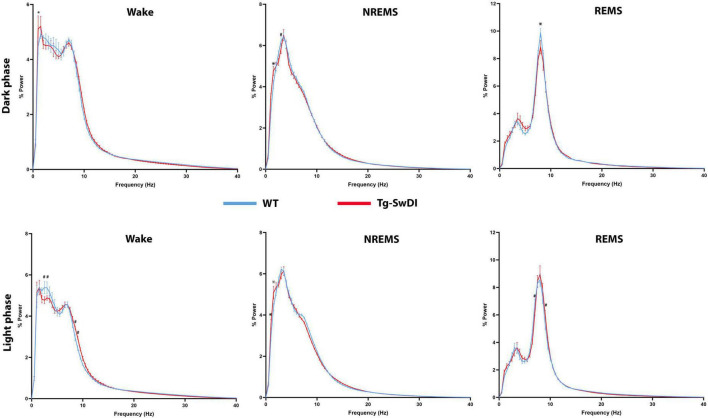
Comparisons of state-dependent power spectra between Tg-SwDI and WT mice. *p < 0.05; #*p* < 0.001.

No significant changes were observed in the amount of time spent in each sleep/vigilance stage during the light (inactive) phase, and the average wake bout length during this phase did not differ between the WT and Tg-SwDI groups. However, EEG power analysis revealed several significant shifts in EEG power spectra during both the dark and light phases (Figure 3).

### A decreased number of orexin immunoreactive cells in 12-month-old Tg-SwDI mice

**FIGURE 4:**
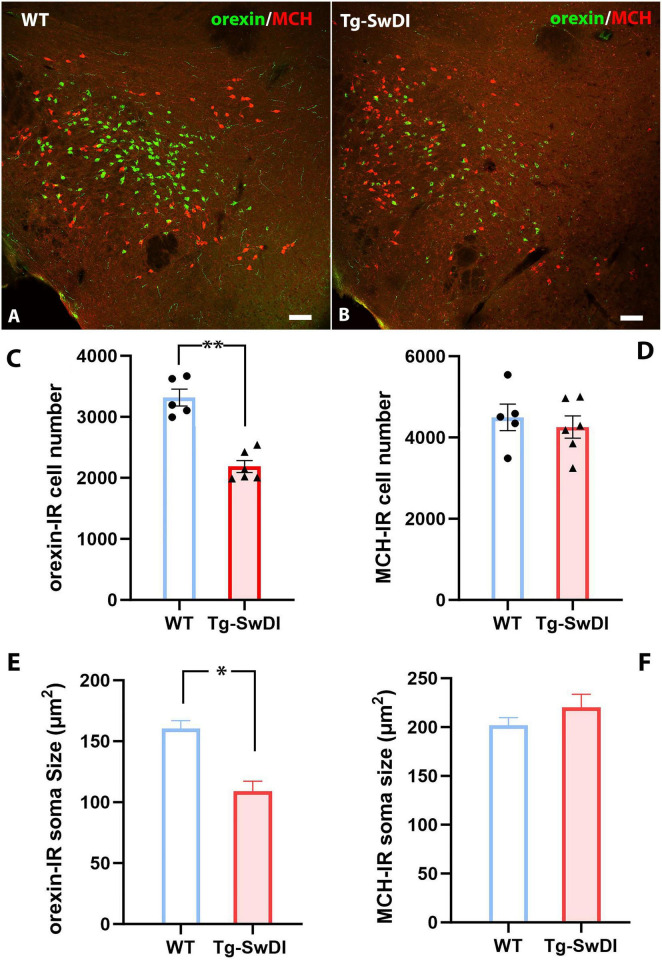
**(A,B)** Representative pictures of orexin-IR (Green) and MCH-IR (Red) neurons in the lateral hypothalamus of a WT **(A)** mouse and a Tg-SwDI mice **(B)**. **(C,D)** Comparisons of orexin-IR and MCH-IR cell numbers between WT and Tg-SwDI group (**p < 0.0001). **(E,F)** Comparisons of orexin-IR and MCH-IR cell soma size (area) between WT and Tg-SwDI mice (*p < 0.001). Scale bars in **(A,B)** = 100 μM.

Since increased NREM sleep, shortened wake bouts, and power spectra shifts are commonly observed in narcoleptic mice with neuropeptide orexin (hypocretin) deficiency or ablated orexin neurons (Chemelli et al., 1999, Chen et al., 2009, Liu et al., 2011, Hung et al., 2020), we questioned whether changes in orexin neurons could underlie these sleep alterations in these Tg-SwDI mice. Therefore, we examined the number of hypothalamic orexin immunoreactive (orexin-IR) and melanin-concentrating hormone-immunoreactive (MCH) immunoreactive (MCH-IR) neurons, two cell groups regulating arousal and sleep in the same brain region (Figures 4A,B). Our analysis revealed a 34% reduction in orexin-IR cells in Tg-SwDI mice compared to WT mice (3318 ± 138.0 in WT versus 2187 ± 97.6 in Tg-SwDI, *p* < 0.0001, *t* = 6.86, *df* = 9) (Figure 4C). Additionally, the average soma size of orexin-IR neurons was significantly smaller in Tg-SwDI (160.4 ± 6.6 μm^2^ in WT versus 109.1 ± 8.1μm^2^ in Tg-SwDI, *p* = 0.001, *t* = 4.76, *df* = 9) (Figure 4E). Although the number of MCH-IR cells showed a slight decrease in Tg-SwDI mice, this difference was not statistically significant (4494 ± 326.8 in WT versus 4257 ± 273.7 in Tg-SwDI, *p* = 0.59, *t* = 0.56, *df* = 9) (Figure 4D) and there was no significant change in the average soma size of MCH-IR cells between the two groups (202.0 ± 7.8 μm^2^ in WT versus 220.1 ± 13.6 μm^2^ in Tg-SwDI, *p* = 0.30, *t* = 1.10, *df* = 9) (Figure 4F). To investigate whether the reduction in orexin-IR cells is specifically linked to vascular amyloidosis, we quantified orexin-IR and MCH-IR cells in age-matched 5xFAD mice, which exhibit exclusively parenchymal amyloidosis. Our analysis showed no significant differences in the numbers of orexin-IR and MCH-IR cells between the WT and 5xFAD groups (Supplementary Figure S3).

**FIGURE 5:**
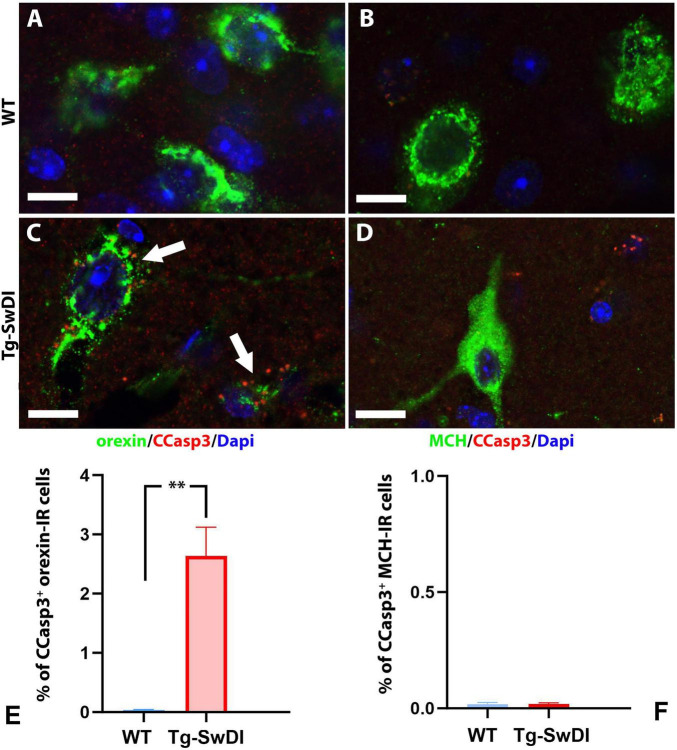
**(A–D)** Representative pictures of CCasp3 expression in orexin-expressing or MCH-expressing neurons in the lateral hypothalamus of a WT **(A,B)** and a Tg-SwDI **(C,D)** mouse. Immunoreactivities of CCasp3 were scarce in WT mice but could be frequently observed in Tg-SwDI mice. Arrows in pane C point to CCasp3^+^ orexin-IR cells. **(E,F)** Comparisons of CCasp3 expression in orexin-IR **(E)** and MCH-IR **(F)** cells between WT and Tg-SwDI mice (**p = 0.0009). Scale bar = 10 μM.

To determine whether cell death or apoptosis contributes to the reduced number of orexin-IR cells in Tg-SwDI mice, we examined the expression of cleaved caspase-3 (Asp175) (CCasp3), a reliable marker for cell apoptosis, in brain sections from WT and Tg-SwDI mice co-stained with orexin or MCH. In WT mice, CCasp3 immunoreactivity was weak across all brain regions, rarely detected in orexin-IR or MCH-IR cells (Figures 5A,B). In contrast, in the Tg-SwDI hypothalamus, Tg-SwDI mice showed significantly elevated CCasp3 expression (*p* = 0.0009, *t* = 4.87, *df* = 9), with approximately 2.6% of orexin-IR cells containing CCasp3 immunoreactivity in the cytoplasm or nucleus (Figures 5C,E). However, CCasp3 immunoreactivity in MCH-IR cells was still rare (Figures 5D,F).

## Discussion

Tg-SwDI mice, originally developed by Dr. Van Nostrand in 2004, are C57BL/6-based transgenic mice expressing the human neuronal amyloid beta-precursor protein (APP) gene harboring Swedish K670N/M671L, Dutch E693Q, and Iowa D694N mutations under the control of the mouse Thy1 promoter (Davis et al., 2004). Adult Tg-SwDI mice exhibited early-onset and robust microvascular Aβ deposits and have been used in many studies as a capillary cerebral amyloid angiopathy (capCAA) model. However, the types (fibrillar or nonfibrillar) and distribution patterns (vascular or parenchymal) of Aβ deposits differed between genotypes in Tg-SwDI mice, with homozygotes showing a more balanced distribution of vascular and parenchymal Aβ deposits/plaques while hemizygotes having more nonfibrillar and vascular Aβ deposits in the brain.

In addition to Aβ accumulation, these mice also show other brain pathological changes, such as neuroinflammation, astrocytosis, and blood-brain barrier (BBB) disruption, which are accompanied by cognitive and memory impairments (Davis et al., 2004, Miao et al., 2005, Fan et al., 2007, Van Vickle et al., 2008, Xu et al., 2014, Jakel et al., 2017, Rosas-Hernandez et al., 2020, Abdallah et al., 2021, Marazuela et al., 2022).

Although sleep disturbances are reported in various Alzheimer’s disease (AD) mouse models, this is the first study to examine sleep patterns in Tg-SwDI mice. Our findings reveal that 12-month-old hemizygous Tg-SwDI mice display increased NREM sleep and fragmented waking during the active phase, along with shifts in power spectra. We also identified a significant reduction in hypothalamic orexin-expressing cells as a potential neural basis for these sleep disturbances.

Although increased sleep or excessive sleepiness can be seen in advanced, late-stage AD, it is uncommon in early- or middle-stage AD patients and young and adult AD mice. For example, 11-12-month-old APP23, J20, APP/PS1, PLB1, and APPNL-G-F knock-in mice exhibit decreased NREM sleep or REM sleep duration/power and increased wakefulness or fragmented NREM sleep (Jyoti et al., 2010, Platt et al., 2011, Van Erum et al., 2019, Filon et al., 2020, Maezono et al., 2020). Similarly, studies on 11–18-month-old 5xFAD and 3xTg-AD mice found no significant differences in sleep durations (Kent et al., 2018, Oblak et al., 2021). Tau models like PS19 (P301S) and rTG4510 mice also show NREM and REM sleep reductions at 11 months (Holth et al., 2017, Holton et al., 2020). A 2023 study by Kam et al., revealed that PS19 mice over 10 months old showed decreased REM sleep and sleep fragmentation. These disruptions were reversed by administering a dual orexin receptor antagonist (DORA), suggesting that an overactive orexin system plays a role in these sleep abnormalities (Kam et al., 2023). CVN-AD mice, a crossbreed of APPSwDI (another name of Tg-SwDI) mice and nitric oxide synthase-2 (NOS2) knockout mice, exhibit both vascular Aβ deposits and phosphorylated tau deposits (Colton et al., 2008, Wilcock et al., 2008). A study from 2021 reported that 9–10-month-old homozygous female CVN-AD mice displayed increased sleep time and fragmented wake/sleep during the dark phase, a pattern resembling what we observed in Tg-SwDI mice. However, this study did not compare sleep patterns with naïve Tg-SwDI mice, leaving gaps in understanding their baseline sleep characteristics (Nwafor et al., 2021).

Intriguingly, the sleep increases and active-phase sleep/wake fragmentation seen in Tg-SwDI mice resemble symptoms observed in narcolepsy, a disorder linked to orexin deficiency or neuronal loss in humans and gene deletion (Chemelli et al., 1999, Nishino et al., 2000, Hungs and Mignot, 2001, Tabuchi et al., 2014). Motivated by these similarities, we examined the orexin neuron status and found that 12-month-old Tg-SwDI mice have a significant reduction in orexin-expressing neurons. Previous studies have noted diurnal fluctuations and age-related decline in orexin cell numbers across species, including humans. In mice, the number of orexin cells is stable in the first 400 days of life and shows marked decline between at 800 days of age (Brownell and Conti, 2010). Severe loss of orexin neurons is also reported in late-stage AD due to widespread neurodegeneration (Kessler et al., 2011, Fronczek et al., 2012, Hunt et al., 2015, McGregor et al., 2017). Given that our study involved adult mice sacrificed at a consistent time (ZT02:00–03:00), the reduced orexin neuron count in Tg-SwDI mice cannot be attributed to diurnal or age-related changes.

Immunohistochemistry (IHC) and immunofluorescence (IF) have been the dominant methods for quantifying neurons or other cells with a specific phenotype. One of the limitations is that cells producing low antigen levels may not be detected in IHC or IF, causing so-called “false-negative” results. It is possible that diurnal fluctuations in orexin levels lead to undetectable orexin-expressing cells in the inactive phase. The same group also identified that opiates increased the number of orexin-expressing neurons in human and mouse brains and later concluded that these increases could be the result of enhanced orexin production in cells having sub-detection levels of orexin rather than the actual loss of orexin-expressing neurons (Thannickal et al., 2018; McGregor et al., 2024). A 2023 study even proposed that orexin deficiency in human narcolepsy could stem from epigenetic silencing of the orexin gene rather than neuron loss (Seifinejad et al., 2023). In contrast, we detected higher-than-normal expression of the cleaved Caspase-3, an apoptosis marker, in orexin-expressing neurons in the Tg-SwDI mice, suggesting that cell death may contribute to the reduction. Other mechanisms, such as epigenetic inhibition, may also play a role in the decreased number of orexin-expressing neurons.

Neuronal loss is a hallmark of AD pathology, though the mechanisms behind it are not yet fully understood (Goel et al., 2022). While transgenic mouse models often fail to replicate significant neuronal loss, some lines do show mild to moderate loss (Eimer and Vassar, 2013, Wirths and Zampar, 2020). Whether Tg-SwDI mice have neuronal loss is still arguable; some studies found that Tg-SwDI mice did not lose neurons unless combined with NOS2 gene knockout or hypertension induction (Wilcock et al., 2008, Van Nostrand et al., 2010, Kruyer et al., 2015). However, a study from 2016 reported the loss of cholinergic neurons in Tg-SwDI mice (Foidl et al., 2016). Our study may be the first to document the selective loss of orexin neurons in the hypothalamus of Tg-SwDI mice.

While neuronal loss often occurs in regions with high Aβ burden, the hypothalamus, the least affected area by Aβ deposits in AD models like 5xFAD and Tg-SwDI mice (Gureviciene et al., 2019, Tsui et al., 2022), may undergo cell loss through a combination of mechanisms such as soluble Aβ toxicity, neuroinflammation, oxidative stress, microbleeds, and compromised neurovascular function (Park et al., 2014, Duncombe et al., 2017, Merlini et al., 2019, Vander Zanden et al., 2019).

Given that the 5xFAD mice, which feature exclusively parenchymal Aβ deposits, do not show significant loss of orexin-expressing neurons in the hypothalamus (Supplementary Figure S3), we suggest that microvascular rather than parenchymal Aβ deposition is a critical factor in this neuronal loss in Tg-SwDI mice. Vascular Aβ deposits may stimulate capillary pericytes to constrict capillary and reduce cerebral blood flow (CBF), reducing glucose/oxygen supplies to local neurons. Orexin neurons may be more susceptible than MCH neurons to the changes caused by microvascular Aβ.

Research has explored the therapeutic potential of orexin receptor antagonists to enhance sleep, reduce Aβ burden, and improve cognitive function in AD (Duncan et al., 2019, Zhou et al., 2020, Lucey et al., 2023). The partial loss of orexin neurons and subsequent increase in sleep in 12-month-old hemizygous Tg-SwDI mice may help limit parenchymal Aβ accumulation. However, this protective effect diminishes with age as Aβ deposit formation becomes more pronounced. Despite its benefits for sleep, reduced orexin function may exacerbate cognitive decline through mechanisms unrelated to sleep (Naumann et al., 2006, Blackwell et al., 2017). Further investigation is needed to fully understand the mechanisms behind orexin neuron loss and its consequences in AD pathology.

Different types of amyloidosis and tauopathy may impact distinct groups of sleep/wake-regulating neurons, leading to specific sleep disturbances in Alzheimer’s disease. Tg-SwDI mice serve as valuable animal models for studying the specific role of microvascular amyloid in sleep regulation and AD pathogenesis. Hemizygous Tg-SwDI mice, at 12 months of age, serve as a model of microvascular amyloidosis, displaying a distinct sleep phenotype featuring increased NREM sleep and fragmented wakefulness during the active phase. The observed reduction in hypothalamic orexin-expressing neurons may underlie these sleep changes. Future research is needed to uncover the mechanisms driving the selective loss of neurons in Tg-SwDI mice and to understand its impact on disease progression.

## Data Availability

The raw data supporting the conclusions of this article will be made available by the authors, without undue reservation.
