# Supplementary material for: Reduction of orexin-expressing neurons and a unique sleep phenotype in the Tg-SwDI mouse model of Alzheimer’s disease
Source: Front Aging Neurosci. 2025 Feb 4;17:1529769. doi: 10.3389/fnagi.2025.1529769 (PMC11832706; doi:10.3389/fnagi.2025.1529769)
Supplement: Supplementary file 1 [file Data_Sheet_1.pdf]

Supplementary Figures:

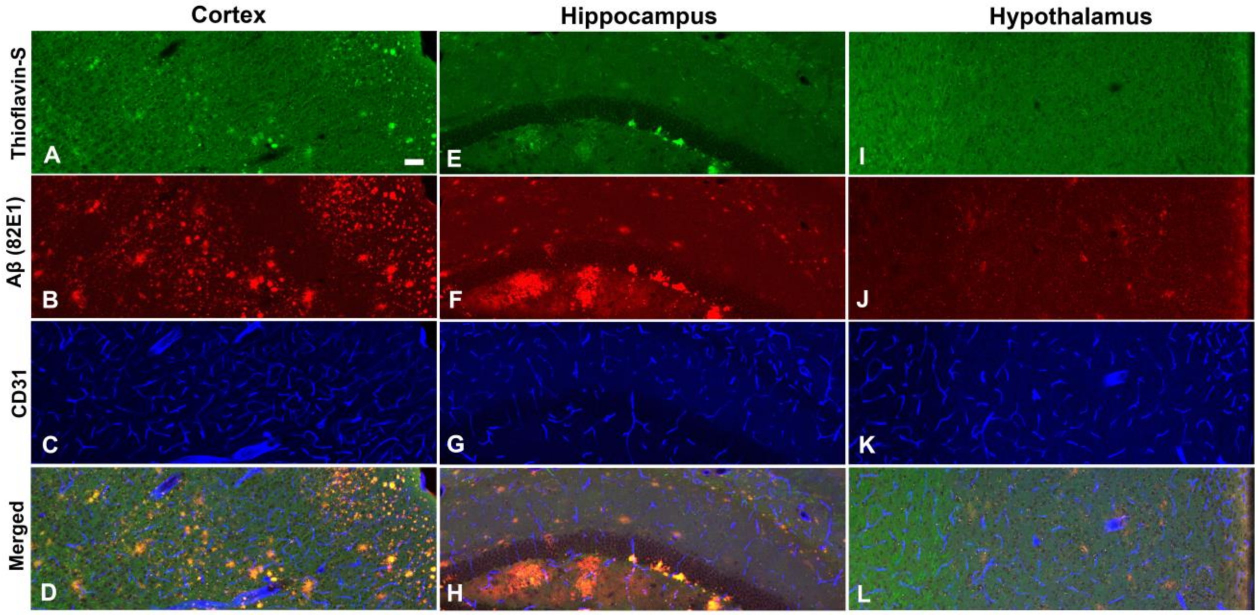

Figure S1. The distribution of Aβ deposits in the cortex (A-D), hippocampus (E-H), and hypothalamus (I-L) of a 12-month-old homozygous Tg-SwDI mouse reveals widespread ThioS$^+$ Aβ plaques (green) across these brain regions, overlapping with 82E1$^+$ Aβ deposits (red). Notably, vascular Aβ deposits are reduced compared to the intense staining observed in parenchymal Aβ deposits. Blue: CD31$^+$ blood vessels. Scale bar = 50 μM.

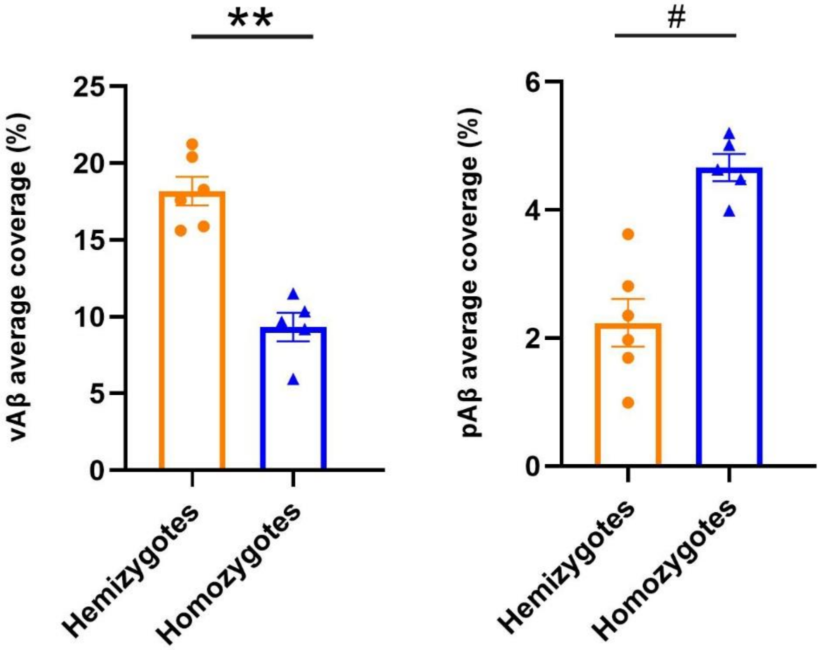

Figure S2. Quantification of vascular Aβ (vAβ) and parenchymal Aβ (pAβ) deposit coverage in 12-month-old hemizygous (n=6) and homozygous (n=5) Tg-SwDI mice was performed. Using NIH ImageJ software, vAβ coverage was calculated as the percentage of blood vessels affected by vAβ (including perivascular Aβ), while pAβ coverage was determined as the percentage of brain parenchyma affected by pAβ, following the method described by (Park et al. 2013). Ten brain sections from each mouse were analyzed, and the values represent the average coverage of the cortex, hippocampus, and lateral hypothalamus. The results indicate that hemizygous mice exhibit significantly greater vAβ coverage (**: $p<0.0001$, $t=6.62$, $df=9$) and lower pAβ coverage (#: $p=0.0005$, $t=5.33$, $df=9$) compared to homozygous mice.

Park, L., J. Zhou, P. Zhou, R. Pistick, S. El Jamal, L. Younkin, et al. (2013). "Innate immunity receptor CD36 promotes cerebral amyloid angiopathy." Proc Natl Acad Sci U S A **110**(8): 3089-3094 DOI: 10.1073/pnas.1300021110.

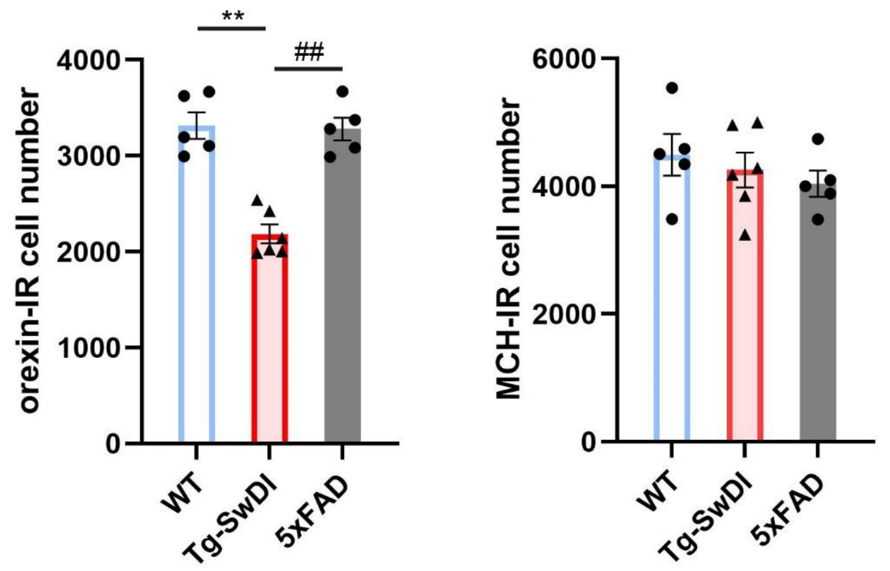

Figure S3. Using the same immunostaining and stereology techniques, orexin-IR and MCH-IR cells were quantified in five 12-month-old 5xFAD mice (both sexes) and compared to WT and Tg-SwDI mice. Statistical analysis was performed using one-way ANOVA followed by Bonferroni's multiple comparisons test (GraphPad Prism 9.2). Significant differences in the number of orexin-IR cells were observed among the three groups ($F_{(2,13)}$=31.77, p<0.0001). The Tg-SwDI group exhibited a significant reduction in orexin-IR cells compared to both the WT (**: p<0.0001, t=6.91, df=13) and 5xFAD groups (##: p<0.0001, t=6.68, df=13), while no reduction was observed between the WT and 5xFAD groups (p>0.99, t=0.22, df=13). No significant differences were detected in the numbers of MCH-IR cells among the three groups.
